# A preliminary survey of the practice patterns of United States *Guild Certified Feldenkrais Practitioners*^CM^

**DOI:** 10.1186/1472-6882-10-12

**Published:** 2010-04-01

**Authors:** Patricia A Buchanan

**Affiliations:** 1 Doctor of Physical Therapy Program, Des Moines University, 3200 Grand Avenue, Des Moines, Iowa, USA

## Abstract

**Background:**

The *Feldenkrais Method*^® ^of somatic education purports to guide people of varying ages and abilities to improve function. Many people choose this method to aid with recovery from injury, manage chronic conditions, or enhance performance even though limited research supporting its safety and effectiveness exists to guide decisions about use and referral. Very little information about practitioner characteristics and practice patterns is publicly available to assist researchers in the design of appropriate safety and effectiveness studies. The purpose of this study was to obtain an initial overview of the characteristics of United States *Guild Certified Feldenkrais Practitioners*^CM^.

**Methods:**

Of 1300 certified *Feldenkrais*^® ^practitioners at the time of the study, there were 1193 practitioners with email accounts who were sent invitations to complete a web-based survey. The survey inquired about practice locations, additional credentials, service patterns and workloads during the previous 3 months. Response rate and descriptive statistics were calculated.

**Results:**

The survey had a 32.3% (385/1193) response rate. The top states in which responders practiced were California (n = 92) and New York (n = 44). Most responders did not hold other credentials as traditional health care providers or as complementary and alternative medicine providers. Among those who did, the most common credentials were physical therapist (n = 83) and massage therapist (n = 38). Just over a third of traditional health care providers only provided *Feldenkrais *lessons, compared to 59.3% of complementary and alternative providers. On average, responders saw 7.6 ± 8.1 (median = 5) clients per week for individual lessons, 8.4 ± 11.5 (median = 5) clients per week for group lessons, and 2.9 ± 3.9 (median = 2) new clients per month for individual lessons.

**Conclusions:**

This preliminary survey of United States *Guild Certified Feldenkrais Practitioners *indicated that most practiced in the west and northeast, did not hold additional credentials, and had part-time practices. Those who were traditional health care providers were more likely than complementary and alternative medicine providers in other areas to combine their services. These results provide a foundation for further analyses of *Feldenkrais *practitioner characteristics and practice patterns that can aid the design of safety and effectiveness studies, and enhance use and referral decision-making.

## Background

The *Feldenkrais Method*^® ^of somatic education is an approach to learning designed to facilitate improved function in daily activities, work and leisure [1,2]. The National Center for Complementary and Alternative Medicine of the United States National Institutes of Health classifies the *Feldenkrais Method *as a movement therapy within the larger category of manipulative and body-based practices [3,4]. Certified practitioners verbally and manually guide clients' movements and direct their attention to various aspects of sensing, feeling, thinking and moving during individual (*Functional Integration*^®^) or group (*Awareness Through Movement*^®^) lessons. As a result, clients presumably refine their ability to make perceptual discriminations that are useful in organizing more efficient movements that can reduce pain, aid recovery from injury, and allow more movement options for meeting functional goals [1]. Overall, clients learn skills that enhance their self-efficacy for managing their health and wellness.

Moshe Feldenkrais, D.Sc. (1904-1984) developed this relatively new method and completed the first United States training of practitioners 32 years ago [5]. To become certified practitioners, persons must complete approximately 800 hours of training typically spread over 4 years. Much of the training involves experiencing many *Awareness Through Movement *and *Functional Integration *lessons to deepen one's understanding of action, perception, emotion, and thought [6]. Midway through the training, participants may begin teaching verbally-guided *Awareness Through Movement *lessons to the public, but must complete the training before providing manually-guided *Functional Integration *lessons [7]. To maintain certification, practitioners must participate in continuing education [8].

Despite limited research into the safety and effectiveness of the *Feldenkrais Method*, thousands of people in the United States [3] and more around the world seek guidance from practitioners to improve function, whether as part of recovering from an injury or illness, managing a congenital or progressive condition, or enhancing performance in sports or the arts. Anecdotal and phenomenological accounts are common in the *Feldenkrais Guild*^® ^of North America's publications, such as *SenseAbility *[9]. Existing peer-reviewed studies vary in quality of evidence and include randomized control comparative effectiveness [10], controlled comparative effectiveness [11,12], randomized crossover [13], randomized control [14], single group [15-18], case series [19], and single case [20] study designs. While limited, these studies lend support for the effectiveness of *Feldenkrais Method *interventions among persons with a variety of health conditions, including work-related injuries [10], multiple sclerosis [13,17], pain [11,16,18], stroke [15,20], and assorted orthopaedic problems [12,14,19].

Unlike other complementary and alternative medicine practitioners, including massage therapists, acupuncturists, and naturopaths [21-24], very little is known about *Feldenkrais *practitioners and their practice patterns. Other than the rare case series, most information about certified practitioners is anecdotal, and limited to what is available through review of the membership directory of the *Feldenkrais Guild *of North America or the practitioner listing on its website [25].

Lack of basic information about *Feldenkrais *practitioners, their practice patterns, and their clients presents an obstacle to the development of research programs that could more rigorously evaluate the safety and effectiveness of the *Feldenkrais Method*. Consumers and health care providers could use such information to make informed decisions about appropriate use of and referral to certified practitioners for *Feldenkrais *lessons. As a first step in removing this obstacle, the purpose of this study was to obtain an initial overview of United States *Feldenkrais *practitioner characteristics. These findings about geographic location, additional credentials, and client visits provide the foundation for establishing more detailed assessments of practice patterns.

## Methods

I designed a web-delivered pilot survey to obtain basic information about practitioner characteristics, including whether practitioners held other traditional or complementary and alternative medicine (CAM) credentials, and to estimate service patterns and workloads during the preceding 3 months. Table 1 presents the survey questions.

**Table 1:** Survey questions

Question
1. In the past 3 months, have you provided *Feldenkrais Method*^® ^lessons to any clients in the United States outside of *Feldenkrais *Training Programs?
2. In what state do you primarily practice as a *Guild Certified Feldenkrais Teacher^®^*?
3. Do you hold a current license or certification as a traditional health care provider? (Note: please do not include massage therapy here.)
4. If you answered yes, please check all that apply. (30 options plus other)
5. Besides your *Feldenkrais*^® ^certification, do you hold a current license or certification as a complementary or alternative health care provider?
6. If you answered yes, please check all that apply. (24 options plus other)
7. Think back over the past 3 months. How much of your *Feldenkrais*^® ^practice is in a setting in which you provided ONLY *Feldenkrais*^® ^lessons (including techniques based on the teachings of Dr. Feldenkrais, such as Bones for Life or Sounder Sleep)?
8. Think back over the past 3 months. How much of your *Feldenkrais*^® ^practice is in a setting in which YOU PROVIDED *Feldenkrais*^® ^lessons in conjunction with another TRADITIONAL form of health care for which you are licensed or certified?
9. Think back over the past 3 months. How much of your *Feldenkrais*^® ^practice is in a setting in which YOU PROVIDED *Feldenkrais*^® ^lessons in conjunction with another COMPLEMENTARY OR ALTERNATIVE form of health care for which you are licensed or certified?
10. On average, how many clients did you see in 1 week for INDIVIDUAL *Feldenkrais*^® ^lessons (*Functional Integration*^® ^or *Awareness Through Movement*^®^)? (Please enter a whole number.)
11. On average, how many clients did you see in 1 week for GROUP *Feldenkrais*^® ^lessons (*Awareness Through Movement*^® ^classes or workshops)? (Please enter a whole number.)
12. On average, how many NEW clients did you see in 1 MONTH for INDIVIDUAL *Feldenkrais*^® ^lessons (*Functional Integration*^® ^or *Awareness Through Movement*^®^)? (Please enter a whole number.)

In April 2009, I verified with the *Feldenkrais Guild *of North America the current number of *Guild Certified Feldenkrais Practitioners*^CM ^(also known as *Guild Certified Feldenkrais Teachers*^®^) in the United States (N = 1300), and obtained the current list of all certified practitioners with email accounts (N = 1193). After securing approval from the Institutional Review Board of Des Moines University, I attempted to contact all listed practitioners by email. Practitioners first received an alert email announcing the survey, followed shortly by more detailed information about the study and an invitation to complete the survey within 2 weeks. After 1 week, nonresponders received a reminder email. Practitioners acknowledged their consent to the study by submitting the survey.

The survey administration tool automatically entered individual responses into Microsoft^® ^Office Excel^® ^2007 spreadsheets. I used my knowledge as a certified practitioner, such as lessons typically last about an hour and clients usually have a series of lessons, to screen data about service profiles for unreasonable/outlier results suggestive of entry error (e.g., entering 80 new individual clients for question 12). I also reviewed responses to questions about additional credentials to eliminate redundant or incorrectly categorized answers (e.g., answering other--massage therapist for question 4). Following data cleaning, I used question-specific response n's (not all responders answered every question) to calculate descriptive statistics within Excel including counts, percentages, means, standard deviations, medians, and ranges. I calculated response rate based on the total number of responders and the number of practitioners with email accounts. As an assessment of the validity of this preliminary survey, I performed non-response analyses by comparing survey respondents with the accessible member database (2009 print directory and on-line directory) for the distributions among states, traditional health care provider credentials, and additional CAM credentials. The member database has limitations because not all members adequately specify other credentials and non-member information is limited to name and year of certification within the print directory.

## Results

### Survey participation

**Figure 1:**
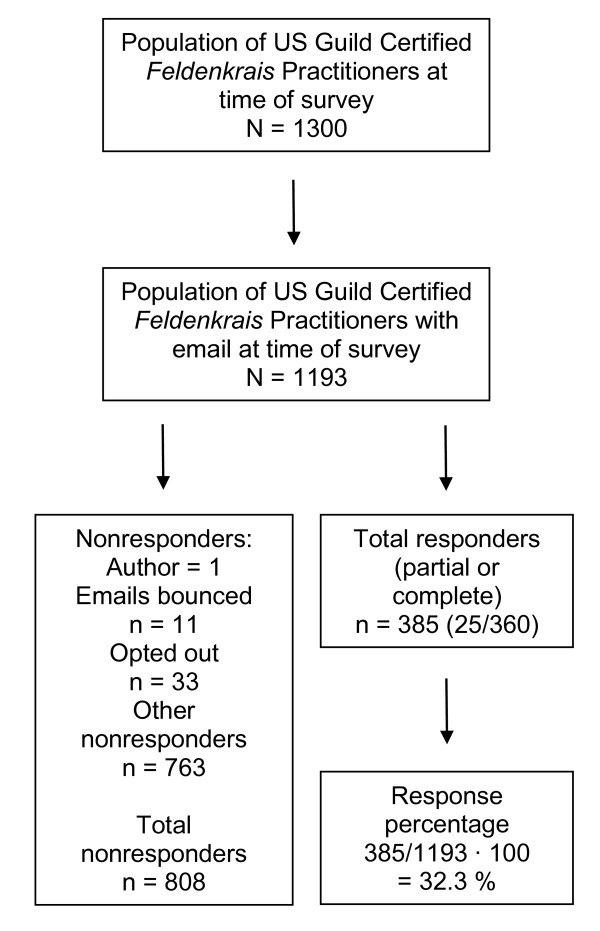
**Population, nonresponder and responder numbers, and survey response percentage**.

Figure 1 presents details about the population, numbers of responders and nonresponders, and response percentage. As the investigator, I opted not to complete the survey.

### Practitioner locations

The highest number of practitioners (n = 92 of 367 responders, 25%) were located in California, followed by New York (n = 44, 12%). Following the region conventions used by the United States Census Bureau, most practitioners were in western states, with more than twice as many practitioners as the next most populated northeast region. Table 2 lists practitioner counts for states within region.

**Table 2:** Numbers of practitioners within regions and by states in order of decreasing frequency.

Northeast region (n = 80)		Midwest region (n = 50)		South region (n = 58)		West region (n = 179)	
**States**	**n**	**States**	**n**	**States**	**n**	**States**	**n**

New York	44	Illinois	14	North Carolina	11	California	92
Pennsylvania	10	Michigan	10	Virginia	10	Washington	23
New Jersey	8	Indiana	6	Maryland	9	Oregon	18
Maine	7	Wisconsin	6	Florida	6	Colorado	13
Connecticut	4	Minnesota	5	Georgia	5	New Mexico	11
Massachusetts	4	Ohio	3	Oklahoma	4	Arizona	9
New Hampshire	2	Iowa	2	Tennessee	4	Utah	6
Vermont	1	Missouri	2	Texas	3	Idaho	4
		Kansas	1	District of Columbia	2	Hawaii	2
		Nebraska	1	South Carolina	2	Alaska	1
				Arkansas	1		
				Kentucky	1		

To check for a possible systematic bias, I examined whether or not a regional imbalance resulted due to missing responses. The regional response rates (number of survey responders/total number of practitioners with email addresses and with known locations) were 34.0% (80/235) in the northeast, 37.3% (50/134) in the midwest, 36.7% (58/158) in the south, and 32.6% (177/543) in the west. These similar response rates indicate there was no regional imbalance. A chi-square test of independence (chi-square = 1.64, df = 3, *p *= 0.65) provided additional support for the absence of regional bias. A state-by-state analysis of the pattern of response rates showed wider variations. However, this was mainly a factor of the small number of potential respondents (members) in some states. The regional rates are considered more stable and therefore reliable indicators of regional variation.

### Additional credentials and their usage

Most *Feldenkrais *practitioners were not additionally licensed or certified as traditional health care providers. Of the 31.7% (116 of 366 responders) who were, the most common credential by far was physical therapist (including Doctor of Physical Therapy; n = 83). The next most frequent professions were occupational therapist (n = 11), nurse (registered nurse or other advanced nursing, n = 7), and certified athletic trainer (n = 4).

By far the majority of the directory members who held traditional health care provider licenses were physical therapists or occupational therapists. These together constituted 85.2% (231/271) of this membership group. The response rate from these two groups was 40.7%, which is somewhat higher than the overall response rate of 32.3%. With the exception of speech and language pathologists who had a response rate of 25% (1/4), the response rates among the other license holders were also higher than the overall average response rate. On this basis it may be argued that the survey responses may be more representative of practitioners who hold traditional licenses than that of the non-traditionally licensed practitioners.

A smaller percentage (23.2%, 85 of 366 responders) of *Feldenkrais *practitioners reported holding licenses or certifications as CAM providers. Massage therapy was the most common practice (n = 38), followed by Reiki (n = 10), yoga (n = 6), qi gong (n = 5), tai chi and meditation (both n = 4).

Among all members with additional CAM credentials, the most common was massage therapy (31%, 57/184). The response rate for massage therapists was 66.7%. Collectively, practitioners with other CAM credentials had a 59.8% (110/184) response rate, well above the overall response rate of 32.3%. As with traditionally licensed practitioners, survey responses may be more representative of practitioners with other CAM credentials than those without these extra certifications or licenses.

Just over half of all responders (52.2%, 190 of 364 responders) reported they provided only *Feldenkrais Method *lessons during their practices. Among those with a traditional health care provider credential, 38.7% (67 of 173 responders) offered only *Feldenkrais Method *lessons in their practices. Conversely, 12.7% (n = 22) always included traditional care with *Feldenkrais Method *lessons. More than half of the practitioners with CAM credentials (59.3%, 89 of 150 responders) reported giving only *Feldenkrais Method *lessons in their practices. Only 2.7% (n = 4) reported always combining *Feldenkrais Method *lessons with other CAM practices.

### Client visits

Practitioners answered 3 questions about client visits during the preceding 3 months. On average, the 353 responders reported seeing 7.6 ± 8.1 clients (median = 5, range 0 to 50) in 1 week for individual lessons. They typically saw 8.4 ± 11.5 clients (median = 5, range 0 to 85) in a week for group lessons. During 1 month, practitioners reported seeing 2.9 ± 3.9 new clients (median = 2, range 0 to 30) for individual lessons.

## Discussion

To my knowledge, this is the first public study of the practice patterns of United States certified practitioners of the *Feldenkrais Method*. Thus, comparisons with other studies are not yet possible. While this is a preliminary survey limited to practitioners with email addresses and does not afford presentation of information suitable for inferential purposes, the study provides previously unavailable information about the characteristics of these CAM practitioners, including their United States locations, provision of other services through additional health care and CAM credentials, and workloads.

The geographic distribution of practitioners that is apparent from this study may reflect the locations of *Feldenkrais *professional training programs (i.e., practitioners are probably concentrated around geographic locations where training outlets are available). For example, Dr. Feldenkrais conducted 2 trainings in the United States prior to his death. The first training in San Francisco, California ended in 1977, and many members of the Amherst, Massachusetts training graduated in 1983. Since that time, numerous trainings have occurred in the west and northeast, with fewer trainings in the midwest and south. Additional research that inquires about practitioners' training programs can clarify the relationship between practitioner locations and training locations.

The majority of responding practitioners did not hold additional credentials as traditional or CAM health care providers. This study did not examine whether practitioners with additional credentials held multiple credentials, but did identify that the most common licenses were as physical therapists and massage therapists. Overall, the majority of practitioners (52.2%) offered only *Feldenkrais *lessons within their practices. This majority held among CAM practitioners (59.3%), but not among those with traditional health care credentials (38.7%). Anecdotally, most clients currently pay directly for *Feldenkrais *lessons. However, practitioners with traditional health care credentials may provide billing to health insurers. One factor that may affect these differing practice patterns could be that clients working with practitioners who hold traditional health care credentials receive additional services that qualify for or are necessary to obtain insurance reimbursement. This premise needs examination with more detailed studies that inquire about clients' methods of payment for services and the actual types of services provided during sessions.

The number of clients that practitioners saw varied widely, ranging on a continuum from some practitioners not currently seeing clients to those with full-time practices. Based on the mean numbers of clients seen for individual (7.6) and group (8.4) lessons per week and new clients for individual lessons per month (2.9), most practitioners apparently had part-time practices. This is comparable to massage therapists who saw 10-15 patients per week [22,24], but distinct from the nearly full-time practices of acupuncturists (27-34 patients per week) and naturopaths (30-33 patients per week) [22].

The results of this preliminary survey have provided basic information about the practice patterns of certified practitioners. Importantly, this study has suggested many more questions for consideration. In addition to practitioners' training locations, payment sources, and details about the services provided within sessions, it would be useful to have more comprehensive information about demographics, practice settings, work characteristics, and reasons for client visits. Clarifying practitioners' interests in participating in clinical/practice-based, applied, or basic research studies would help research planning. More detailed and objective information about practice patterns and interest in collaborative research should assist researchers to identify key questions and focus their resources more efficiently as they design studies to assess the safety and effectiveness of the *Feldenkrais Method*.

One strength of this study is that it is likely the first open assessment of the practice characteristics of *Feldenkrais Method *practitioners in the United States. Invitations went to all certified practitioners with email addresses comprising the vast majority of all practitioners. Regional response rates were similar to the overall response rate which supports a valid geographical representation.

There are limitations to this study reflective of its preliminary nature. I did not formally assess the validity of the survey questions. Many respondents volunteered suggestions that will assist in the design of subsequent studies that will include validation of questions. Another limitation is that a small percentage of certified practitioners (8.2%, 107/1300) were excluded from the opportunity to participate in the study because they did not have email addresses. Future study methodologies will add mechanisms to include these certified practitioners. An interesting response bias exists in the present study: practitioners with traditional or CAM licenses or certifications were more likely to respond to the survey than those without additional credentials. So, although balanced regionally, the results are likely more representative of practitioners with added credentials. Finally, the response rate of 32.3% may pose an overall risk of lower accuracy for the survey findings.

## Conclusions

This preliminary survey of United States *Guild Certified Feldenkrais Practitioners *indicated most practiced in the west and northeast. While the majority did not hold other credentials, the most common traditional health care providers were physical therapists, and the most common CAM providers were massage therapists. Among practitioners with additional credentials, traditional health care providers were more likely than CAM providers to combine their services. Client visits suggested most practitioners had part-time practices. These novel findings can assist development of more detailed studies of practice patterns that can guide the design of safety and effectiveness studies. In turn, consumers and health care providers should have more evidence to decide to use or refer to *Feldenkrais *practitioners.

## Competing interests

The author is a *Guild Certified Feldenkrais Teacher *and member of the *Feldenkrais Guild *of North America (FGNA). She has received small honoraria from FGNA for presenting workshops at its 2005 and 2009 annual conferences.

## Pre-publication history

The pre-publication history for this paper can be accessed here:

http://www.biomedcentral.com/1472-6882/10/12/prepub
